# Rehabilitation and return to sport criteria following surgical treatment of osteochondritis dissecans of the capitellum: a systematic review

**DOI:** 10.1016/j.jseint.2023.11.003

**Published:** 2023-11-30

**Authors:** Andrew George, Brendan M. Holderread, Brian M. Phelps, Emily R. Erwin, William Singer, Robert A. Jack

**Affiliations:** Houston Methodist Orthopedics & Sports Medicine Houston, Houston, TX, USA

**Keywords:** Elbow, Capitellum, Osteochondritis dissecans, Rehabilitation, Return to sport, Protocol

## Abstract

**Background:**

Osteochondritis dissecans (OCD) of the capitellum is a well-described condition that most commonly affects adolescent throwing athletes and gymnasts. There is no gold standard rehabilitation protocol or timing for return to sport (RTS) after surgical management of OCD of the capitellum.

**Hypothesis/Purpose:**

The purpose of the study was to identify in the existing literature any criteria used for RTS following surgical treatment of OCD of the capitellum. The hypothesis was that surgeons would utilize length of time rather than functional criteria or performance benchmarks for RTS.

**Methods:**

Level 1 to 4 studies evaluating athletes who underwent surgery for OCD of the capitellum with a minimum follow-up of 1-year were included. Studies not describing RTS criteria, including less than 1-year follow-up, non-operative management only, and revision procedures were excluded. Each study was analyzed for RTS criteria, RTS rate, RTS timeline, sport played, level of competition, graft source (if utilized), and postoperative rehabilitation parameters. Assessment of bias and methodological quality was performed using the Coleman methodology score and RTS value assessment.

**Results:**

All studies reported a rehabilitation protocol with immobilization followed by bracing with progressive range of motion. RTS rate was 80.9% (233/288). The majority of studies reported using time-based criteria for RTS (11/15). The most commonly reported timeline was 6 months (range: 3-12 months).

**Conclusion:**

The overall RTS rate after surgical treatment of capitellar OCD is high with no consensus on RTS criteria. The two most consistent RTS criteria reported in the literature are return of elbow range of motion and healing demonstrated on postoperative imaging. There is a wide range of time to RTS in the literature, which may be sport dependent. Further research is needed to develop functional and performance-based metrics to better standardize RTS criteria and rehabilitation protocols.

Osteochondritis dissecans (OCD) of the capitellum is a well-described condition that most commonly affects adolescent throwing athletes and gymnasts.^11^ It is theorized to result from repetitive valgus compression and shear forces across the radio-capitellar joint. This leads to localized injury and vascular insufficiency to the capitellum, and ultimately separation of articular cartilage from subchondral bone.^5^

Stable OCD lesions of the capitellum can be treated non-operatively with activity restriction and close observation.^19^ Stable lesions that do not respond to nonoperative care, as well as unstable lesions, warrant surgical management.^23^ Surgical management is diverse and may include open or arthroscopic débridement, loose body removal, microfracture, or drilling.^6^ Fixation (cartilage repair) may be warranted for larger lesions that are not completely displaced. In cases of larger lesions that engage the radial head, as well as revision cases in which arthroscopic treatment has failed, osteochondral autograft or allograft transplantation surgery may be warranted.^22^

While surgical techniques and outcomes following operative management of capitellar OCD lesions have been investigated in depth in the literature, the timeline of rehabilitation and return to sport (RTS) criteria after surgery is not standardized. The purpose of this study was to identify any described criteria in the literature used to advance patients through their rehabilitation and ultimately to RTS. The authors hypothesized that length of time from surgery would be used most often, rather than functional criteria or performance benchmarks. This hypothesis was based on several recent studies in the sports and shoulder/elbow literature that found time-based criteria were most often used after other surgeries of the shoulder and elbow, including surgical stabilization for anterior shoulder instability and ulnar collateral ligament reconstruction.^9^^,^^12^

## Materials and methods

### Search strategy

A search was conducted in PubMed and Embase databases from their inception to December 2022 using the terms “elbow”, “arthroscopy”, “athlete”, “return to”, “osteochondritis dissecans”, “treatment”, “capitellar” or “capitellum”, and “outcome”. All references of articles selected were evaluated to identify articles that were potentially missed by this search strategy.

**Figure 1:**
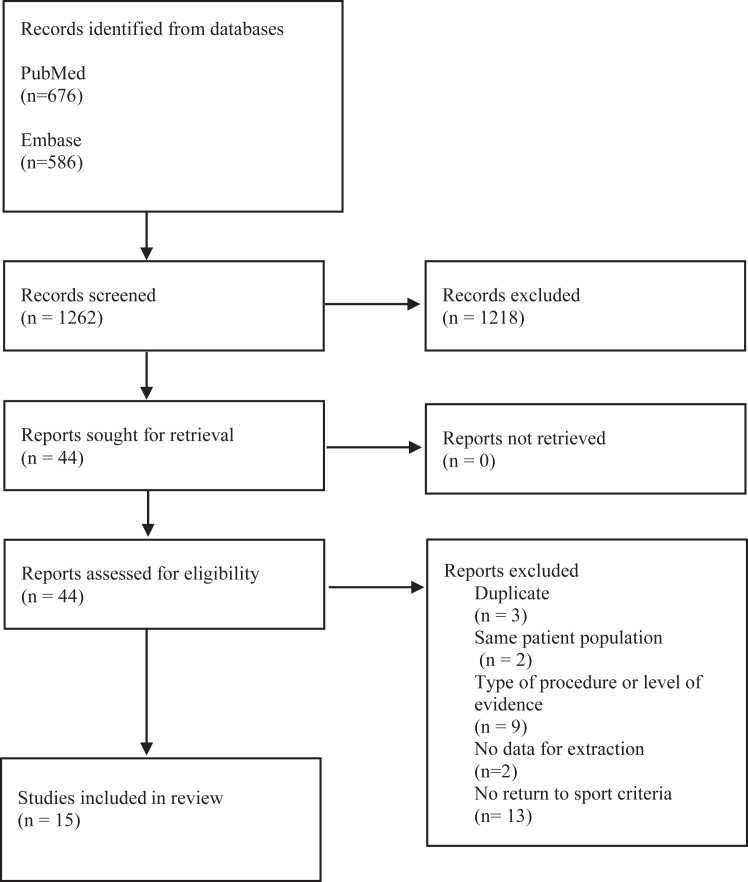
PRISMA (Preferred Reporting Items for Systematic Reviews and Meta-Analyses) flow diagram depicting assessment strategy for including studies in the systematic review.

The article search and review were completed by 3 reviewers under supervision of an attending orthopedic surgeon who has fellowship training in sports medicine. The Preferred Reporting Items for Systematic Reviews and Meta-Analyses flowchart provides the exact number of articles identified, screened, excluded, and included with rationale (Fig. 1). Preferred Reporting Items for Systematic Reviews and Meta-Analyses criteria were followed throughout the described systematic review.

### Inclusion/exclusion criteria

Included studies were those (1) evaluating patients undergoing surgical treatment of OCD of the capitellum, (2) with a minimum 1-year follow-up, and (3) with an English version of the article available for review. Studies not describing RTS criteria, narrative reviews, technical notes/tips, letters to the editor, studies with less than 1-year follow-up, studies describing nonoperative management only, and studies evaluating only revision procedures, were excluded.

### Patient demographics and outcomes

Surgical indication, surgical technique, total numbers of patients, elbows receiving operative treatment, age of patient cohort (mean), level of competition, and sport played were extracted for evaluation. Patient-reported outcomes surveys were not recorded due to heterogeneity in surveys used. However, mean time of follow-up and success in return to activities or sport were extracted. Particular attention was given to analyzing explicit criteria utilized in decision making for gradual RTS/activity or full and unrestricted RTS/activity. Articles with missing or unclear data were excluded.

### Quality of literature assessment

Coleman Methodology Scores (CMS) were assigned by two independent reviewers. CMS quantitatively assesses methodological quality of a reported research study with 10 criteria providing a possible score from 0 to 100 (Table I). The assessment specifically evaluates study size, follow-up, surgical procedures, type of study, diagnostic certainty, description of surgical technique, description of postoperative rehabilitation, outcome criteria, procedure for assessing outcome, and description of subject selection process.

**Table I:** Coleman Methodology Scores along with the means and standard deviations for each scoring category with a maximum score of 100.

Authorship	1	2	3	4	5	6	7	8	9	10	Total
Ayzenberg et al, 2021^2^	7	5	10	0	5	5	10	10	5	5	62
Allahabadi et al, 2020^1^	10	2	7	0	5	5	10	2	3	5	49
Matsuura et al, 2020^16^	10	5	10	0	0	5	5	3	3	5	46
Matsuura et al, 2017^15^	10	5	10	0	5	5	10	2	3	5	55
Oshiba et al, 2016^20^	4	5	10	0	5	5	10	2	4	5	50
Maruyama et al, 2016^14^	4	5	10	0	3	5	10	2	3	5	47
Takeba et al, 2015^24^	10	2	10	0	0	5	10	2	3	5	47
Koehler et al, 2015^13^	0	5	10	10	5	5	10	2	3	5	55
Uchida et al, 2015^25^	10	5	10	10	5	5	10	3	5	5	68
Nishinaka et al, 2014^17^	10	5	10	10	5	5	10	2	5	5	67
Wulf et al, 2012^27^	7	5	10	0	5	5	10	2	3	5	52
Bojanić et al, 2012^7^	4	5	10	10	0	5	10	2	5	5	56
Rahusen et al, 2006^21^	0	5	10	10	0	3	0	2	5	5	40
Yamamoto et al, 2006^28^	10	5	10	10	5	5	0	2	3	5	55
Byrd et al, 2002^8^	4	5	0	0	0	3	10	2	3	5	32
Mean ± SD	6.3 ± 3.9	4.4 ± 1.2	8.8 ± 3.0	4.0 ± 4.9	3.3 ± 2.4	4.8 ± 0.8	8.3 ± 3.5	3.0 ± 2.4	4.1 ± 1.6	5.3 ± 1.3	52.1 ± 9.5

*SD*, standard deviation.

1, study size (10); 2, mean follow-up (5); 3, No. of interventions per group (10); 4, study type (15); 5, diagnostic certainty (5); 6, description of surgical technique (5); 7, description of postoperative rehabilitation (10); 8, outcome criteria (10); 9, procedure for assessing outcomes (15); 10, description of patient selection process (15).

### Quality of return to sport assessment

Quality of RTS methodology was assessed according to methodology described by Zaman et al^29^ by two independent reviewers (Table II). This assessment evaluates description of rehabilitation protocol, timeline for return to activity, objective/subjective criteria, and specific measurement criteria.

**Table II:** Scoring report for return to sport value assessment.

Surgical technique	Authorship	Rehabilitation protocol	Timeline for return to activity	Objective/Subjective criteria	Specific measurement criteria	RTS article rating score (4)
Arthroscopic microfracture/debridement	Allahabadi et al, 2020^1^	1	1	1	1	4
	Wulf et al, 2012^27^	1	1	1	0	3
	Bojanić et al, 2012^7^	1	1	0	0	2
	Rahusen et al, 2006^21^	1	0	0	0	1
	Matsuura et al, 2020^16^	1	1	0	0	2
	Byrd et al, 2002^8^	1	1	1	0	3
Osteochondral autograft transplantation	Oshiba et al, 2016^20^	1	1	1	0	3
	Maruyama et al, 2016^14^	1	1	1	1	4
	Matsuura et al, 2017^15^	1	1	1	1	4
	Nishinaka et al, 2014^17^	1	1	1	1	4
	Yamamoto et al, 2006^28^	1	1	1	0	3
	Ayzenberg et al, 2021^2^	1	1	1	0	3
Arthroscopic fragment fixation	Allahabadi et al, 2020^1^	1	1	1	1	4
	Takeba et al, 2015^24^	1	1	1	0	3
	Koehler et al, 2015^13^	1	1	1	0	3
	Uchida et al, 2015^25^	1	1	1	1	4
	Mean ± SD	1.0 ± 0.00	0.9 ± 0.26	0.8 ± 0.41	0.3 ± 0.49	3.1 ± 0.88

*RTS*, return to sport.

A maximum of 1 point was given per category if the study met the criteria.

### Statistical analysis

All data were collected and analyzed using Microsoft Excel (Microsoft Corp., Redmond, WA, USA).

## Results

1262 studies identified were assessed for eligibility. Fifteen studies met inclusion criteria with 288 athletes.^1^^,^^2^^,^^7^^,^^8^^,^^13^^,^^14^, ^15^, ^16^, ^17^^,^^20^^,^^21^^,^^24^^,^^25^^,^^27^^,^^28^ The methodological quality was fair (CMS: 52.1+/− 9.5) with most studies reporting sufficient follow-up, as well as good diagnostic certainty and description of surgical technique (Table I). Surgical procedures included arthroscopic microfracture/débridement, arthroscopic fragment fixation, or osteochondral autograft transplantation. Among grafts used in the osteochondral autograft transplantation cohort, the most common site of graft harvest was the ipsilateral olecranon. Baseball was the most commonly identified sport (13/15, Table III). Recreational/amateur level of preoperative play was most commonly reported (6/15, Table IV).

**Table III:** The distribution of sports included in the overall patient population.

Surgical technique	Sport included in patient population	No. of studies included
Arthroscopic microfracture/debridement	Not reported	2
	Baseball	3
	Softball	1
	Gymnastics	3
	Basketball	1
	Tennis	2
	Water polo	1
	Handball	1
	Track and field	1
	Karate	1
	Volleyball	1
Osteochondral autograft transplantation	Not reported	1
	Baseball	6
	Basketball	2
	Soccer	1
	Gymnastics	1
	Tennis	1
	Weightlifting	1
Arthroscopic fragment fixation	Not reported	1
	Baseball	3
	Gymnastics	1
	Lacrosse	1

**Table IV:** Levels of play at time of surgery.

Surgical technique	Level of play before surgery	No. of studies included
Arthroscopic microfracture/debridement	Not reported	2
	Recreational	2
	Middle school	3
	High school	3
	Competitive/elite	1
Osteochondral autograft transplantation	Not reported	1
	Recreational	4
	Competitive/elite	2
Arthroscopic fragment fixation	Not reported	2
	Recreational	1
	Competitive/elite	1

Several studies described multiple patient populations.

Described rehabilitation protocols were extracted, and recurring themes are summarized in Table V, stratified by type of surgical procedure. All studies reported a rehabilitation protocol with immobilization followed by bracing with progressive range of motion (ROM). The majority of studies reported using time-based criteria for RTS (11/15). Twelve out of fifteen studies described a multiphase rehabilitation program, with an average of 4 phases. Ten studies utilized a postoperative survey. The most commonly used survey was the Timmerman-Andrews assessment. All studies reported RTS as a rate. The overall RTS rate was 80.9% (233/288). The most commonly reported time to RTS was 6 months (4/15). The earliest time to RTS was 3 months, and the longest time was 12 months (Table VI).

**Table V:** Summary of the postoperative rehabilitation parameters.

Surgical technique	Postoperative rehabilitation parameters	No. of studies included
Arthroscopic microfracture/debridement	Immobilization/bracing with progressive ROM/continuous passive motion	5
	Strengthening	3
	Sport-specific protocol	4
	Supervised physical therapy	5
	Multiphase program	5
	# of phases	3.6∗
	Throwing mechanic education	1
	Interval throwing program	2
	Postop survey used for RTS criteria	3
Osteochondral Autograft Transplantation	Immobilization/bracing with progressive ROM/continuous passive motion	6
	Strengthening	4
	Sport-specific protocol	5
	Supervised physical therapy	3
	Multiphase program	5
	# of Phases	4.3∗
	Interval throwing program	6
	Throwing mechanic education	1
	Provided interval throwing program specifics	3
	Postop survey used for RTS criteria	5
Arthroscopic Fragment Fixation	Immobilization/bracing with progressive ROM/continuous passive motion	4
	Strengthening	4
	Sport-specific protocol	1
	Supervised physical therapy	1
	Multiphase program	2
	# of Phases	3∗
	Interval throwing program	1
	Cryotherapy & electrical stimulation	1
	Cardiovascular training	1
	Postop survey used for RTS criteria	3

∗ Average rather than count.

**Table VI:** Return to sport timelines.

Surgical technique	RTS timeline reported	No. of studies included
Arthroscopic microfracture/debridement	N/R	4
	4.5 months	1
	5 months	1
Osteochondral autograft transplantation	N/R	1
	3 months	1
	6 months	3
	8-12 months	1
Arthroscopic fragment fixation	N/R	3
	6 months	1

*RTS*, return to sport.

Studies that only mentioned return to activity or throwing are not represented and are included under the “Not reported” category.

Each article was rated on a scale of 0-4 to assess its value in reviewing RTS criteria as described by Zaman et al^29^ (Table II). All studies reported at least 1 of the following 4 metrics of RTS criteria: rehabilitation protocol, timeline for return to activity, objective/subjective criteria, and specific measurement criteria. The average RTS value score was 3.1 +/− 0.88. Five out of fifteen studies achieved a value of 4/4, which is notably higher than other RTS criteria literature, ie, UCL reconstruction.^12^ Four of these five studies utilized either x-ray or magnetic resonance imaging to confirm graft healing or fragment fixation healing. Three of the five studies utilized return of normal elbow ROM as an objective criterion for RTS. Three of the five studies utilized time-based criteria in addition to these objective measures, ranging from 2-3 months postoperatively before throwing activities resumed.

## Discussion

While surgical techniques and outcomes after operative management of capitellar OCD lesions are well-described, validated rehabilitation protocols and RTS criteria are lacking in the literature. The authors’ hypothesis was supported, as the majority of studies utilized time-based criteria for return to activity, rather than functional criteria or performance-based metrics.

All studies reported a rehabilitation protocol with immobilization followed by bracing with progressive ROM or continuous passive motion. While return to play criteria are more standardized after other sports medicine procedures such as anterior cruciate ligament reconstruction,^3^^,^^4^^,^^10^ there is a lack of well-defined objective criteria that could advance a patient through rehabilitation after capitellar OCD surgery.

Of the 5 studies that achieved 4/4 in terms of their value in assessing RTS criteria as defined by Zaman et al,^8^ the most common objective measures were return of normal elbow ROM without pain and healing on radiographs and/or magnetic resonance imaging. These were also the two most common objective measures utilized when assessing all 15 studies. Objective measures were most often used in conjunction with time-based criteria. While radiographic healing may be one of the more objective measures utilized, this could be limited by the degree of intrarater and inter-rater agreement in assessing OCD characteristics and healing.^18^ Nevertheless, return of elbow ROM and healing on imaging are the two objective measures most consistently found in the literature. Interestingly, no two studies employed the same RTS/activity criteria, highlighting the variability in postoperative protocols. Given this wide variability, further comparative research is needed to determine an optimal protocol and RTS criteria following surgical treatment of capitellar OCD.

In terms of RTS rate, 81% of patients were able to RTS at the same level. There was a wide variety of timelines for RTS, ranging from 3 to 12 months. The average time to RTS was 6 months. This is consistent with previously published work, reporting about an 86% RTS rate at 6 months after surgical management of capitellar OCD lesions.^26^ In a systematic review and meta-analysis of RTS after surgical management of capitellar OCD, Westerman et al^26^ found a greater percentage of athletes were able to return to high-level athletics after osteochondral autograft or allograft transplantation surgery procedures compared to débridement or fixation procedures. However, rehabilitation protocols and RTS criteria were not assessed.

This study has several important limitations. Physicians who regularly treat OCD lesions of the elbow may have published RTS criteria and rehabilitation protocols available for patients and physical therapists. This review would have excluded these protocols in an effort to remain standardized and in accordance with prior RTS criteria study methodology.^12^ Second, while we stratified our data by type of procedure performed, within this stratification there is still likely variability in size and geography of lesion that could affect postoperative rehabilitation protocol and outcomes. Finally, there was variability in the competitive level of athletes. Higher level athletes likely had a more personalized and sport-specific protocol than recreational athletes.

## Conclusion

The overall RTS rate after surgical treatment of capitellar OCD is high with no consensus on RTS criteria. The two most consistent RTS criteria reported in the literature are return of elbow ROM and healing demonstrated on postoperative imaging. There is a wide range of time to RTS in the literature, which may be sport dependent. Further research is needed to develop functional and performance-based metrics to better standardize RTS criteria and rehabilitation protocols.

## Disclaimers:

Funding: No funding was disclosed by the authors.

Conflicts of interest: The authors, their immediate families, and any research foundation with which they are affiliated have not received any financial payments or other benefits from any commercial entity related to the subject of this article.
